# Two-dimensional Hyper-branched Gold Nanoparticles Synthesized on a Two-dimensional Oil/Water Interface

**DOI:** 10.1038/srep06119

**Published:** 2014-08-26

**Authors:** Yonghee Shin, Chiwon Lee, Myung-Seok Yang, Sunil Jeong, Dongchul Kim, Taewook Kang

**Affiliations:** 1 Department of Chemical and Biomolecular Engineering, Sogang University, Seoul, 121-742, Korea; 2 Department of Mechanical Engineering, Sogang University, Seoul, 121-742, Korea; 3 These authors contributed equally to this work.

## Abstract

Two-dimensional (2D) gold nanoparticles can possess novel physical and chemical properties, which will greatly expand the utility of gold nanoparticles in a wide variety of applications ranging from catalysis to biomedicine. However, colloidal synthesis of such particles generally requires sophisticated synthetic techniques to carefully guide anisotropic growth. Here we report that 2D hyper-branched gold nanoparticles in the lateral size range of about 50 ~ 120 nm can be synthesized selectively on a 2D immiscible oil/water interface in a few minutes at room temperature without structure-directing agents. An oleic acid/water interface can provide diffusion-controlled growth conditions, leading to the structural evolution of a smaller gold nucleus to 2D nanodendrimer and nanourchin at the interface. Simulations based on the phase field crystal model match well with experimental observations on the 2D branching of the nucleus, which occurs at the early stage of growth. Branching results in higher surface area and stronger near-field enhancement of 2D gold nanoparticles. This interfacial synthesis can be scaled up by creating an emulsion and the recovery of oleic acid is also achievable by centrifugation.

The unique physical and chemical properties of two-dimensional (2D) metallic nanoparticles have attracted significant interest as promising candidates for various applications including catalysis^1^,^2^, solar-energy harvesting^3^, electronics^4^, metamaterials^5^,^6^,^7^ and biomedicine^8^,^9^,^10^. So far, 2D nanoparticles have been prepared on solid substrates by micro/nanofabrication^11^,^12^. Their applications have, however, been limited by the number of particles and the need for a solid substrate. Colloidal synthesis of these particles requires delicate control over reaction conditions such as chemicals, reaction time and temperature^13^,^14^,^15^,^16^,^17^,^18^,^19^,^20^. Therefore, colloidal synthesis typically proceeds either via temperature control, over a long reaction time, through multiple reaction steps, or with the aid of structure-directing agents such as surfactants or a sacrificial template, which often makes reproduction and scaling up difficult. In particular, branched structures such as metallic nanodendrimers, have been considered difficult to produce and only relatively large dendrimers (> 200 nm) have been synthesized with the aid of a sacrificial graphene oxide^20^.

Interestingly, here we demonstrate that two-dimensional immiscible oil/water interfaces can provide diffusion-controlled growth conditions, leading to the fast formation of 2D hyper-branched gold nanoparticles at room temperature without structure-directing agents.

A small nucleus grows on an oleic acid/water interface into a nanodendrimer, and further structural evolution from dendrimer to urchin takes place while the thicknesses of these nanoparticles remains nearly unchanged, confirming that the nanoparticles on the interface are 2D (Fig. 1a). The morphological transition to nanourchin is completed within a few minutes. The experimental data for the 2D branching of the nucleus at the early stage of growth is consistent with simulations based on the phase field crystal model.

In addition to the aforementioned advantage of having a fast reaction time, no requirement for structural directing agents and room temperature synthesis, the interfacial synthesis of 2D gold nanoparticles has more practical implications (Fig. 1b). The synthesis can be scaled up by creating an emulsion, allowing the production of a large amount of colloidal particles in a short time. Oleic acid is biocompatible and can be easily recovered due to the different densities of oil (*i.e.*, oleic acid) and water after the interfacial synthesis. 2D hyper-branched gold nanoparticles can show a large surface area that is available to neighboring molecules without diffusion limitation, strong near-field enhancement and tunable far-field response. The size can be as small as *ca.* 50 nm, which is well suited for *in vivo* applications.

## Results

### Liquid/liquid interfacial synthesis of sub-100 nm two-dimensional hyper-branched gold nanoparticles

**Figure 1:**
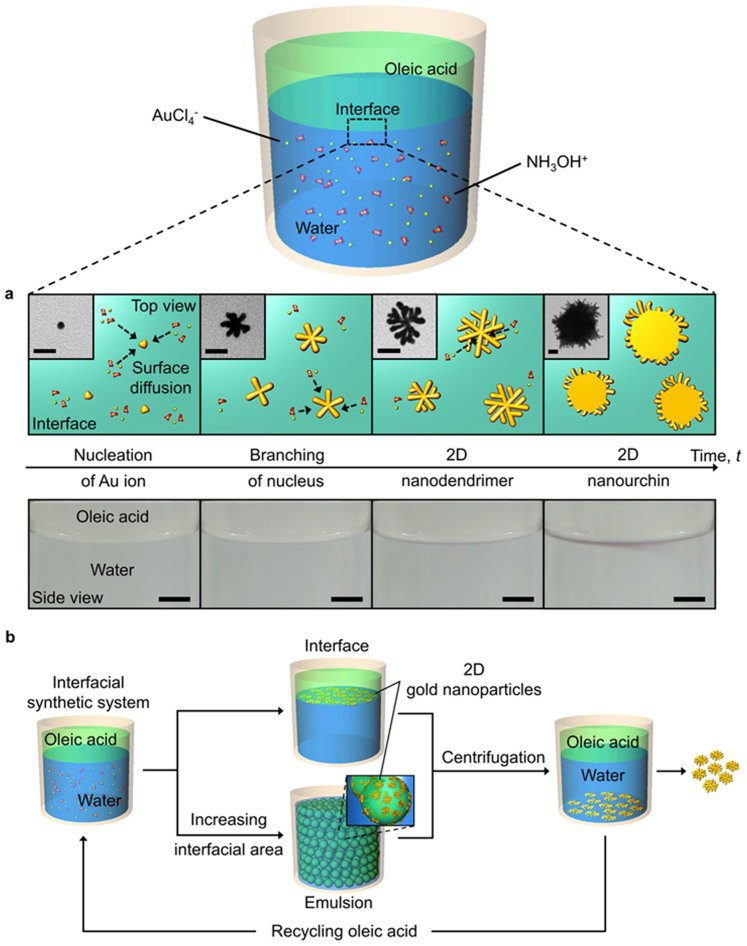
Schematic illustrations and an experimental demonstration for our oil/water interfacial synthesis of two-dimensional (2D) gold nanoparticles. (a), Schematic representation and time-resolved optical images of an oleic acid/water interface for the synthesis of 2D gold nanoparticles (b), Schematic illustration of the interfacial synthesis for recycling of the biocompatible oleic acid and scaling up (creating an emulsion). Scale bars are 25 nm (transmission electron microscopy (TEM) images in (a)) and 5 mm (optical images in (a)), respectively.

Schematic illustrations and an experimental demonstration for our liquid/liquid interfacial synthesis of two-dimensional (2D) gold nanoparticles are presented in Fig. 1 and Supplementary Movie 1, respectively. Briefly, a gold precursor (HAuCl_4_·3H_2_O, tetrachloroauric acid trihydrate) was dissolved in deionized water, followed by the addition of a reducing agent (NH_2_OH·HCl, hydroxylamine hydrochloride) into the aqueous solution. Oleic acid was then slowly poured on top of the solution. Approximately 4 min after the addition of the oleic acid, the liquid/liquid interface appeared light grey in color and gradually changed to pink (Fig. 1a). On the other hand, both water and oil layers remained colorless for over 30 min, indicating that the formation of the nanoparticles selectively takes place at the interface.

**Figure 2:**
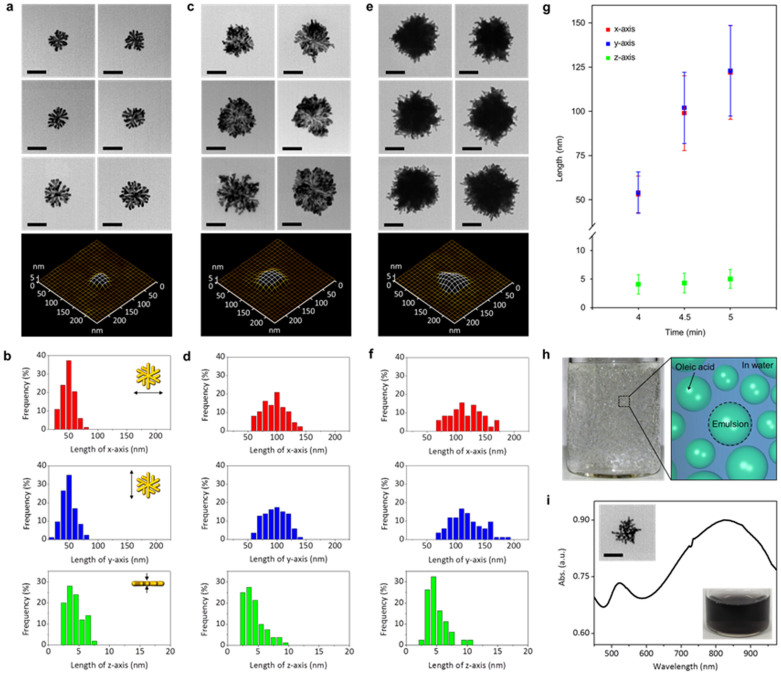
Morphological and optical characterizations of 2D hyper-branched gold nanoparticles. (a), (c), (e), Representative TEM and atomic force microscopy (AFM) images of 2D gold nanoparticles sampled at the interface at (a) 4 min, (c) 4 min 30 s, and (e) 5 min, respectively. (b), (d), (f), Size distributions with respect to x- (red), y- (blue) and z-axes (green) of the particles. (g), Average lengths with respect to x- (red), y- (blue) and z-axis (green) of the particles with increasing reaction time. (h), Optical image and corresponding cartoon of the interfacial synthesis via the oleic acid-in-water emulsion. (i), Representative UV-vis spectrum of the gold nanodendrimer synthesized at the oleic acid-in-water emulsion (top inset: TEM image of the nanoparticle, bottom inset: optical image of the solution). For statistical analyses, 80 particles are randomly selected (Supplementary Fig. 1 and Supplementary Fig. 2). Scale bar is 50 nm.

In order to characterize the gold nanoparticles formed on the interface in detail, the oleic acid/water interface was sampled for transmission electron microscope (TEM) and atomic force microscope (AFM) measurements. To statistically analyze their morphological and structural transitions, 80 particles from each microscope image were randomly selected (Supplementary Fig. 1 and Supplementary Fig. 2). Fig. 2a shows representative TEM and AFM images of the gold nanoparticles collected from the interface at the early stage of growth (at 4 min after the addition of oleic acid). Clearly, the gold nanoparticles show a dendritic morphology along with primary and secondary branches (Fig. 2a and Supplementary Fig. 1). The TEM images show that small gaps in the range of 2–8 nm exist between branches. Average lengths with respect to x, y, and z axes of the particles were 53.5 ± 10.4 nm, 54.2 ± 11.7 nm, and 4.21 ± 1.70 nm, respectively (Fig. 2b and Fig. 2g), indicating that the gold nanoparticles are two-dimensional. Note that the number of secondary branches is dependent on their lateral size (*i.e.*, more secondary branches were observed for larger lateral sizes) while that of primary branches is similar irrespective of the size (Supplementary Fig. 3). The width of both the primary and secondary branches is about 7 nm.

As the reaction time proceeds, the small gaps between the branches are quickly filled with gold atoms and new branches are formed simultaneously (Fig. 2c and Supplementary Fig. 1). After 5 min, the morphology of the particles changed from nanodendrimer to nanourchin with many smaller branches (4–7 nm) on the outer surface (Fig. 2e and Supplementary Fig. 1). The lengths of the nanourchin, with respect to x, y, and z axes were found to be 122.0 ± 26.5 nm, 123.0 ± 25.7 nm, and 5.03 ± 1.65 nm, respectively (Fig. 2f and Fig. 2g). During this morphological transition, lateral sizes with respect to x and y axes increased by about 140% while the thickness (*i.e.*, along z axis) remained nearly constant. This suggests that the gold nanoparticles formed on the oleic acid/water interface are likely to grow along the interface, maintaining their two-dimensional structure. Note that as the reaction time further increased, the lateral sizes of the nanoparticles increased linearly to over 215 nm and the branches disappeared simultaneously. The thickness slightly increased to about 17 nm (Supplementary Fig. 1d).

Since the reducing agent and oleic acid are ionizable depending on solution pH, we examined the effect of solution pH on the formation of gold nanoparticles on the interface. The reaction batches were prepared separately by varying pH from 3.05 to 6.28 (Supplementary Fig. 7). As solution pH was further decreased (*i.e.*, more acidic than pH 3.36), a color change was not observed at the oleic acid/water interface. At higher pH, the color change was observed not only at the interface but also in solution. The gold nanoparticles synthesized at higher pH were no longer two-dimensional. Based on these experimental observations, the interfacial reaction mechanism can be estimated as follows. As gold precursor (HAuCl_4_) dissolves in water, the pH is decreased to 3.36. The reducing agent, hydroxylamine (NH_2_OH) is likely to be protonated to NH_3_OH^+^ since the pK_a_ of NH_2_OH is around 5.9. Due to the weaker reducing capability of NH_3_OH^+^ compared to NH_2_OH, the reduction of the Au ion is strongly limited in solution. However, at the oleic acid/water interface, the carboxylic head group of the oleic acid would assist in the reduction of Au ions, allowing both the formation of a small gold nucleus at the interface^21^,^22^ and the growth along the interface.

### Scaling up the interfacial synthesis by creating an emulsion

Even though our 2D gold nanoparticles undergo a structural transition from nanodendrimer to nanouchin, the reaction intermediate, for example, a gold nanodendrimer can also be selectively obtained by either increasing the interfacial area or decreasing the concentration of the gold precursor. The oleic acid-in-water emulsion was produced by stirring the solution at a moderate speed (Fig. 2h and see Methods section for the detailed preparation method). The stirring speed dictates the size of the emulsion. The gold nanoparticles were collected by centrifugation and re-dispersed in water. As shown in Fig. 2i, the final solution is dark grey in color. The absorbance spectrum of the solution exhibits two distinct surface plasmon resonance (SPR) bands at 525 and 828 nm (Fig. 2i). Two SPR bands in the visible and near-IR range can be attributed to the shape anisotropy (*i.e.*, two-dimensional structure) of the particles^23^,^24^,^25^. TEM images of the particles clearly show the dendritic morphology with primary and secondary branches, which is the same as that of the particles obtained on the planar oleic acid/water interface (Top inset of Fig. 2i and Supplementary Fig. 8). In addition, the thicknesses of these particles were measured by AFM. The average thickness is found to be 7.03 ± 1.12 nm, indicating that the particles obtained from the oil-in-water emulsion are 2D (Supplementary Fig. 9). Note that the selectivity of the nanodendrimer over unwanted by-products such as gold spherical nanoparticles is also dependent on the stirring speed.

### The crystallographic analysis and phase field crystal simulation of 2D gold nanodendrimers

**Figure 3:**
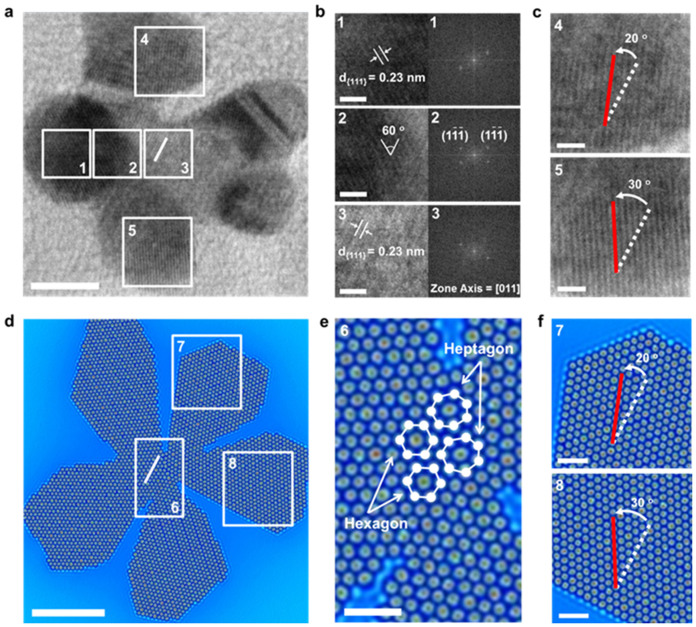
Crystallographic analysis and phase field crystal simulation of 2D gold nanoparticles. (a), High-resolution TEM image of 2D nanodendrimer obtained at the early stage of growth. Crystal lattice of the core (marked with white line) is set as a reference to compare the orientation. (b), Lattice-resolved images and electron diffraction patterns taken along the [011] zone axis from the core to branches (numbered 1-3 boxes in (a)). (c), Magnified images (numbered 4,5 boxes in (a)) showing tilted crystal lattice orientations between the core (white dotted line) and each branch (red line). (d), Phase field crystal model to simulate the early stage of growth. (e), The appearance of non-hexagonal packing of surface Au atoms between the core and branch (numbered 6 box in (d)). (f), Magnified simulation images (numbered 7,8 boxes in (d)) for tilted crystal lattice orientations. Scale bars are 5 nm (a, d) and 1 nm (b, c, e, f), respectively.

Fig. 3a shows representative high-resolution TEM (HRTEM) images of 2D gold nanodendrimers obtained at the early stage of growth. Lattice characteristics of (111) planes were observed from both the core and a branch of the particle (Fig. 3b and Supplementary Fig. 10). Interestingly, tilted crystal lattice orientations between the core and each branch were observed (Fig. 3c). The phase field crystal simulation also produces the branching and tilted crystal lattice orientations which match well with the experimental observation (Fig. 3d, Fig. 3f and Supplementary Movie 2). Furthermore, the crystal growth simulation results suggest that the tilted crystal orientation angles would result from the appearance of non-hexagonal packing of Au atoms (*e.g.*, heptagonal packing) (Fig. 3e).

### Electromagnetic field simulation and Surface-enhanced Raman spectroscopy (SERS) measurements

**Figure 4:**
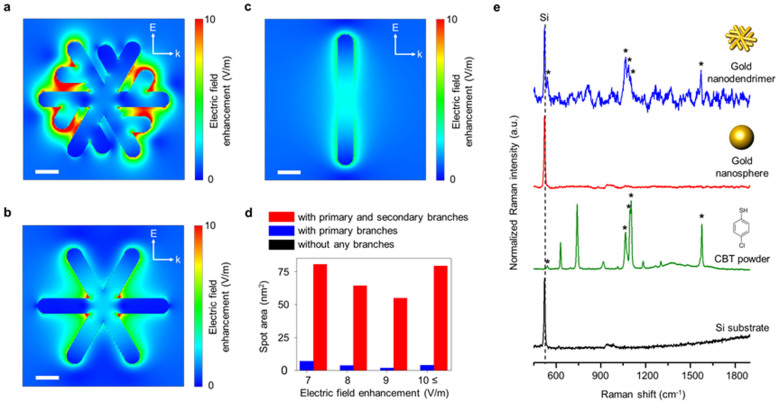
Electromagnetic (EM) simulation of 2D gold nanoparticles and surface-enhanced Raman spectroscopy (SERS) experiment with the 2D gold nanodendrimer. (a–c), Electric field enhancement for 2D gold nanoparticles with (a) primary and secondary branches, (b) primary branches only, without (c) any branches. Scale bar corresponds to 10 nm. (d), Plot of hot spot area of 2D gold nanoparticles with the enhancement factor larger than 7 (V/m). (e), SERS spectra when using colloidal gold nanodendrimer solution (blue line) and 20 nm gold nanospheres solution (red line). Raman spectra of 4-chlorobenzenethiol (CBT) powder (green line) and silicon substrate (black line). The Raman transitions of CBT are marked with asterisks (542, 1066, 1086, 1102, and 1571 cm^−1^, respectively).

Next, electromagnetic field (EM) simulation was carried out using the finite-difference time-domain (FDTD) method (see Supplementary Methods for the detailed simulation). The EM simulation results for three kinds of 2D gold nanoparticles (*i.e.*, 2D gold nanoparticles with primary and secondary branches, with primary branches, and without any branches) are shown in Fig. 4a–c. The number of areas for strong EM enhancement (hot spot) increases with branching (Fig. 4d). Strong EM enhancement is generated in small gaps between branches.

In order to realize the benefit of our 2D hyper-branched gold nanoparticles, 2D gold nanodendrimers, as a proof-of-concept, were tested for surface-enhanced Raman spectroscopy (SERS). 4-chlorobenzenethiol (CBT) was selected as a standard molecule for SERS. For the control experiment, gold nanospheres (GNS), 20 nm in diameter (Supplementary Fig. 12) were tested to compare the SERS signal under the experimental conditions since GNS has a similar amount of Au atoms to the gold nanodendrimer (Supplementary Table 1). From ICP-MS analysis, the concentrations of each nanoparticle were measured to be the same (Supplementary Table 1). Fig. 4e shows normalized SERS spectra of CBT from a colloidal gold nanodendrimer and GNS solutions. No apparent Raman transition is found in the Raman spectrum taken from colloidal GNS (red line in Fig. 4e). In contrast, characteristic Raman transitions of CBT (assigned by asterisks, blue line in Fig. 4e) are clearly observed from colloidal gold nanodendrimer solutions. This SERS enhancement from gold nanodendrimers can be attributed to many factors including a large accessible surface area without diffusion limitation, many hot spots between branches, and the wavelength of the laser. Several studies are currently underway to address each effect in detail but are beyond the scope of this study.

## Discussion

In conclusion, we have demonstrated interfacial synthesis that lead to the fast formation of two-dimensional gold nanoparticles at room temperature without structure-directing agents. The small gold nucleus with a diameter of about 4 nm selectively grows on the interface into a nanodendrimer due to branching at an early stage of growth, which is consistent with numerical simulations based on the phase field crystal model. Gold nanodendrimers further undergo the transition to a larger gold nanourchin as the reaction time proceeds. During this structural transformation, interestingly, the thickness of these nanoparticles is maintained at around 5 nm, indicating that they are two dimensional. The synthesis method described in this paper does not require structural directing agents, has a fast reaction time, consumes less energy, uses a biocompatible and recyclable solvent and is scalable for production. From a material standpoint, we expect that these 2D gold nanoparticles have a substantial impact on catalysis, biomedicine and other areas of research due to their high surface area without diffusion limitation and high packing density, tunable size and excellent near and far-field optical properties.

## Methods

### Synthesis of two-dimensional (2D) gold nanoparticles at an oleic acid/water interface

Two-dimensional gold nanoparticles were synthesized in a 30 ml glass vial. 0.850 ml hydrogen tetrachloroaurate(III) hydrate solution (HAuCl_4_·3H_2_O, 1.2 mg/ml) was mixed with 12.8 ml DI water, and 37.5 *μ*l of aqueous hydroxylamine hydrochloride (NH_2_OH·HCl, 0.05 M) was added to the solution. After homogeneous mixing, 2.8 ml of oleic acid was slowly introduced to form the oleic acid-water interface. Within a few minutes, color was observed at the interface after the addition of oleic acid. 1 ml of the aqueous phase just below the oleic acid-water interface was collected using a pipette at 4 min, 4 min 30 s, 5 min, 6 min and 7 min.

### Synthesis of 2D gold nanoparticles in oleic acid-in-water emulsions

1.53 ml of hydrogen tetrachloroaurate(III) hydrate solution (HAuCl_4_·3H_2_O, 0.715 mg/ml) was diluted with 162.27 ml DI water in a 1-neck round-bottom flask, followed by the addition of 450 *μ*l of hydroxylamine hydrochloride aqueous solution (NH_2_OH·HCl, 0.05 M) with stirring. Under vigorous stirring, 33.6 ml of oleic acid was quickly added to the solution to form oleic acid-in-water emulsions. The reaction in the emulsion mixture was allowed to proceed for 20 min while it was stirred continuously with a magnetic stirrer. After 30 s the stirring was stopped and the mixture was separated into water and oleic acid, and 150 ml of the aqueous phase was collected. The synthesized nanoparticles in the aqueous solution were isolated by centrifugation (5000 rpm, 10 min) and subsequently re-dispersed in water. 20 *μ*l of colloidal gold nanodendrimer solution was dropped onto a carbon-coated 300 mesh TEM grid (Inc. Ted Pella) and the UV-VIS extinction spectra were taken on a JASCO V530 spectrophotometer.

### Growth mechanism simulation via the phase field crystal model

We used a phase field crystal model to simulate the growth of a 2D gold nanodendrimer^26^,^27^,^28^,^29^. This model relies on the dimensionless density, *φ* = (*ρ* − *ρ_ref_*)/*ρ_ref_*, where *ρ* and *ρ_ref_* are the time averaged particle density and a reference solution of particle density, respectively. The detailed explanation for the growth mechanism simulation is included in the Supplementary Methods.

### Particle preparation and functionalization for SERS measurements

For SERS measurements, the gold nanodendrimer and gold nanosphere were functionalized with a 10 mM ethanolic solution of 4-chlorobenzenethiol for 3 h under magnetic stirring at room temperature. Functionalized particles were re-dispersed in water after being centrifuged three times (8000 rpm, 10 min) in order to eliminate the remaining ethanol and 4-chlorobenzenethiol. The concentrations of functionalized gold nanodendrimer and gold nanospheres in solution, obtained by ICP-MS, were converted to the number per unit volume for each sample.

### Raman and SERS measurements

A Raman spectrometer QE65000 from Ocean Optics Inc. and 785 nm laser module I0785MM0350MS from Innovative Photonic Solution Inc. were used for Raman and SERS measurements. Raman measurements were carried out for 4-chlorobenzenethiol powders, 10 mM ethanolic solution of 4-chlorobenzenethiol, ethanol solutions, and the silicon substrate. The Raman measurement was conducted using a 785 nm laser at a power of 250 mW and an integration time of 10 s. SERS measurements were conducted with 50 *μ*l of functionalized gold nanodendrimers and 50 *μ*l of functionalized gold nanospheres on a silicon substrate. The SERS measurement also used a 785 nm laser at a power of 250 mW and an integration time of 10 s. The baseline of the SERS spectrum was corrected before normalization.

## Author Contributions

T.K. conceived this concept. Y.S., C.L. and T.K. designed the experiments. Y.S., C.L. and S.J. performed the experiments. M.Y and D.K. contributed to simulations. Y.S., C.L. and T.K. wrote the manuscript. All authors discussed the results and commented on the manuscript.

## Supplementary Material

Supplementary Information Supplementary Information

Supplementary Information Supplementary Movie 1

Supplementary Information Supplementary Movie 2
