# Supplementary material for: Two-dimensional Hyper-branched Gold Nanoparticles Synthesized on a Two-dimensional Oil/Water Interface
Source: Sci Rep. 2014 Aug 26;4:6119. doi: 10.1038/srep06119 (PMC4143794; doi:10.1038/srep06119)
Supplement: Supplementary Information [file srep06119-s1.pdf]

Supplementary Information for

Two-dimensional Hyper-branched Gold Nanoparticles Synthesized on a Two-dimensional Oil/Water Interface

Yonghee Shin, Chiwon Lee, Myung-Seok Yang, Sunil Jeong, Dongchul Kim, Taewook Kang*

*To whom correspondence should be addressed. e-mail: twkang@sogang.ac.kr

This PDF file includes:

Supplementary Materials and Methods
Supplementary Fig. 1 to 14
Supplementary Table 1 to 2
Captions for Supplementary Movie 1 to 2

Other Suplementary Materials for this manuscript includes the following:

Supplementary Movie 1 to 2

Supplementary Materials and Methods

Chemicals and Materials

All chemicals including Hydrogen tetrachloroaurate(III) hydrate ($HAuCl_4$•$3H_2O$, 99.999%), Hydroxylamine hydrochloride (NH_2OH•HCl, 99.9999%), 4-Chlorobenzenethiol (CBT, 97%) and Oleic acid (90%) were purchased from Sigma Aldrich Inc. and without further purification. Gold nanospheres were purchased from BBI Solutions. Deionized (DI) water with a resistivity of 18 M Ω-cm was used in all cases. All glassware was treated with a piranha solution (H_2SO_4: H_2O_2 = 7:3 v/v, this solution is a very harmful and strong acidic oxidant) for 30 min, and rinsed with DI water for several times.

Synthesis of two-dimensional (2D) gold nanoparticles at an oleic acid/water interface

2D gold nanoparticles were synthesized in a 30 ml glass vial. 0.850 ml hydrogen tetrachloroaurate(III) hydrate solution ($HAuCl_4$•$3H_2O$, 1.2 mg/ml) was mixed with 12.8 ml DI water, and 37.5 μl of aqueous hydroxylamine hydrochloride (NH_2OH•HCl, 0.05 M) was added to the solution. After homogeneous mixing, 2.8 ml of oleic acid was slowly introduced to form the oleic acid-water interface. Within a few minutes, color was observed at the interface after the addition of oleic acid. 1 ml of the aqueous phase just below the oleic acid-water interface was collected using a pipette at 4 min, 4 min 30 s, 5 min, 6 min and 7 min.

Characterization of 2D gold nanoparticles

The morphological properties of the synthesized 2D gold nanoparticles were characterized by transmission electron microscopy (TEM) and atomic force microscopy (AFM). 20 μl of each solution was dropped onto a carbon-coated 300 mesh TEM grid (Inc. Ted Pella) and allowed to remove the solution by using filter paper within 30 s. TEM images were obtained with a JEOL JEM 1010 electron microscope operating at an acceleration voltage of 80 kV. AFM images were obtained with a SPA-400 (Seiko Instrument, Japan).

High resolution TEM analysis of 2D gold nanodendrimer

High resolution TEM studies were performed in a JEOL JEM 3010 electron microscope operating at an acceleration voltage of 300 kV. TEM samples were prepared by carefully dropping 20 μl of dispersion collected at 4min onto a carbon-coated 300 mesh TEM grid (Inc. Ted Pella).

Synthesis of 2D gold nanoparticles in oleic acid-in-water emulsions

1.53 ml of hydrogen tetrachloroaurate(III) hydrate solution ($HAuCl_4$•$3H_2O$, 0.715 mg/ml) was diluted with 162.27 ml DI water in a 1-neck round-bottom flask, followed by the addition of 450 μl of hydroxylamine hydrochloride aqueous solution (NH_2OH•HCl, 0.05 M) with stirring. Under vigorous stirring, 33.6 ml of oleic acid was quickly added to the solution to form oleic acid-in-water emulsions. The reaction in the emulsion mixture

was allowed to proceed for 20 min while it was stirred continuously with a magnetic stirrer. After 30 s the stirring was stopped and the mixture was separated into water and oleic acid, and 150 ml of the aqueous phase was collected. The synthesized nanoparticles in the aqueous solution were isolated by centrifugation (5000 rpm, 10 min) and subsequently re-dispersed in water. 20 μl of colloidal gold nanodendrimer solution was dropped onto a carbon-coated 300 mesh TEM grid (Inc. Ted Pella). TEM images were obtained with a JEOL JEM 1010 electron microscope operating at an acceleration voltage of 80 kV and the UV-VIS extinction spectra were taken on a JASCO V530 spectrophotometer.

Growth mechanism simulation

We used a phase field crystal model to simulate the growth of 2D gold nanodendrimer. This model relies on dimensionless density, $\varphi = (\rho - \rho_{ref}) / \rho_{ref}$, where ρ and ρ_{ref} are time averaged particle density and a reference solution of particle density , respectively. The dynamical evolution is expressed by

$$\frac{\partial \varphi}{\partial t} = \nabla^2 [\{\mu + (1+\nabla^2)^2\}\varphi + \varphi^3] + \alpha' \nabla N$$

, where μ is the phenomenon constant. The term $\alpha' \nabla N$ is the Gaussian noise represented by reaction fluctuation, and α' and N are the noise amplitude and Gaussian random number, respectively. The parameters used are μ = -0.75, α' = 0.00004, and φ_o (initial concentration) = -0.50320. In our simulation, the whole domain size is 2000 x 2000. The equations are discretized in both space and time, and we used Δx = Δy = 1 and Δt = 0.25.

Near-Field Calculation

The near-field optical properties were calculated using a commercially available finite-difference time-domain (FDTD) package (OptiFDTD 8.0). The simulation has used the Drude-Lorentz model which is adopted to investigate the metallic dispersion. The permittivity was set to the values of bulk gold, and we assumed that the 2D gold nanoparticles are embedded in surrounding medium of air. The 785 nm laser source (plane wave, amplitude: 1V/m) is used to excite the particle. The direction of 785 nm laser source is from left to right. The mesh size and simulation space volume are chosen so that further changes in them do not affect the simulation results.

Particle Preparation and functionalization for SERS measurements

For SERS measurements, the gold nanodendrimer and gold nanosphere were functionalized with a 10 mM ethanolic solution of 4-chlorobenzenethiol for 3 h under magnetic stirring at room temperature. Functionalized particles were re-dispersed in water after being centrifuged thrice (8000 rpm, 10 min) in order to eliminate the remaining ethanol and 4-chlorobenzenethiol. The concentrations of functionalized gold nanodendrimer and gold nanospheres in solution, obtained by ICP-MS, were converted to the number per unit volume for each sample.

Raman and SERS measurements

A Raman spectrometer QE65000 from Ocean Optics Inc. and 785 nm laser module I0785MM0350MS from Innovative Photonic Solution Inc. were used for Raman and SERS measurements. Raman measurements were carried out for 4-chlorobenzenethiol powders, 10 mM ethanolic solution of 4-chlorobenzenethiol, ethanol solutions, and the silicon substrate. The Raman measurement was conducted using a 785 nm laser at a power of 250 mW and an integration time of 10 s. SERS measurements were conducted with 50 μl of functionalized gold nanodendrimers and 50 μl of functionalized gold nanospheres on a silicon substrate. The SERS measurement also used a 785 nm laser at a power of 250 mW and an integration time of 10 s. The baseline of the SERS spectrum was corrected before normalization.

a

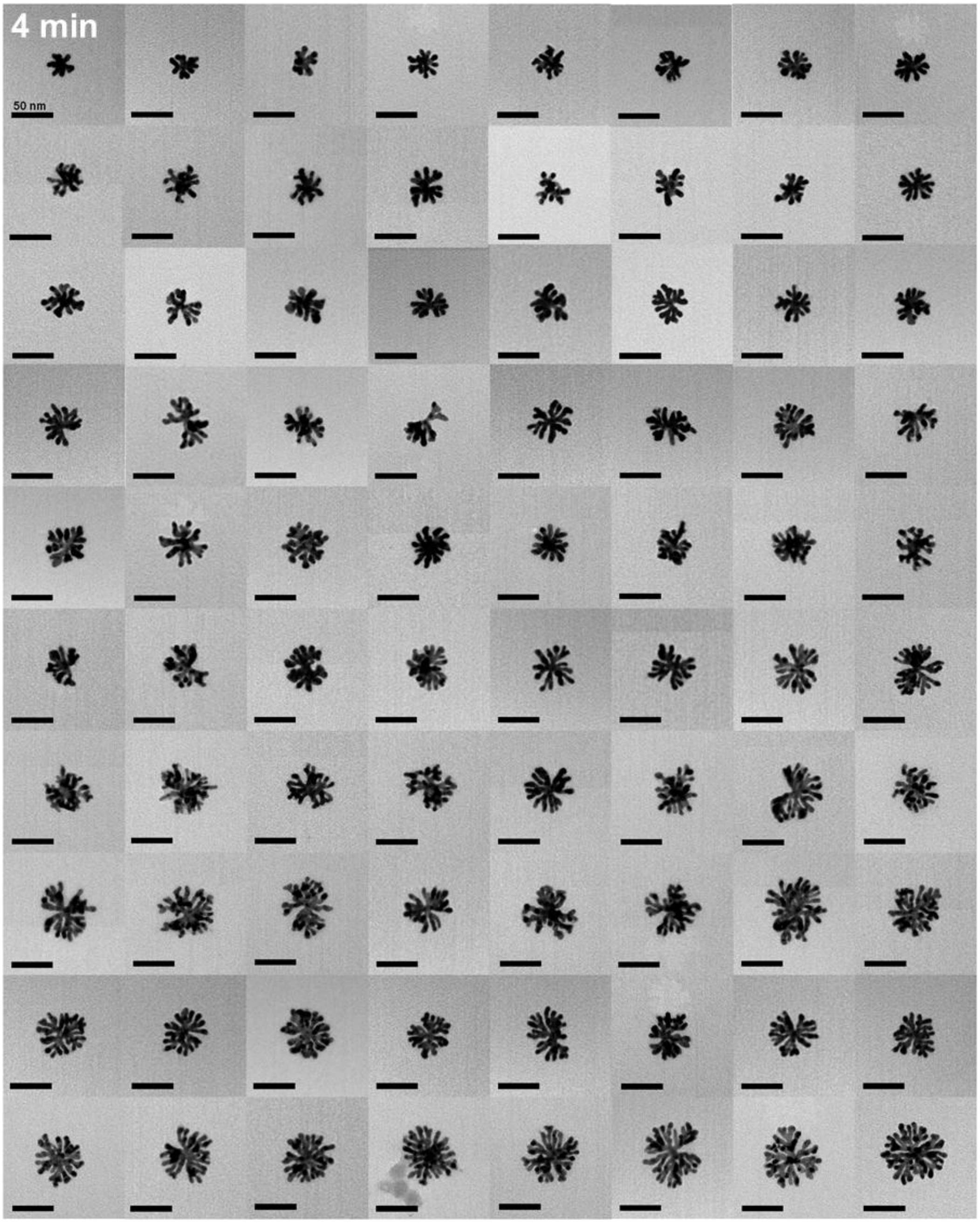

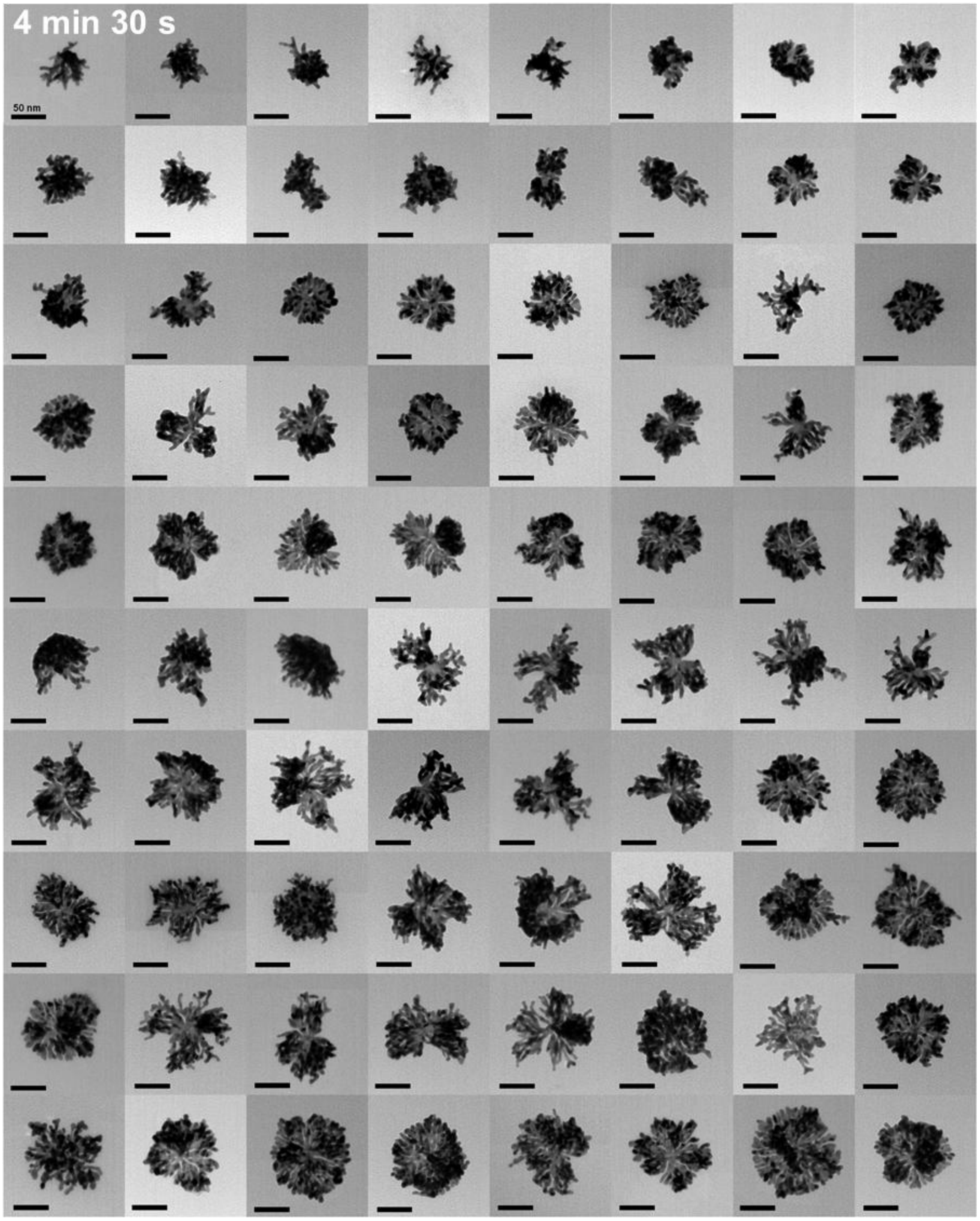
4 min 30 s
50 nm

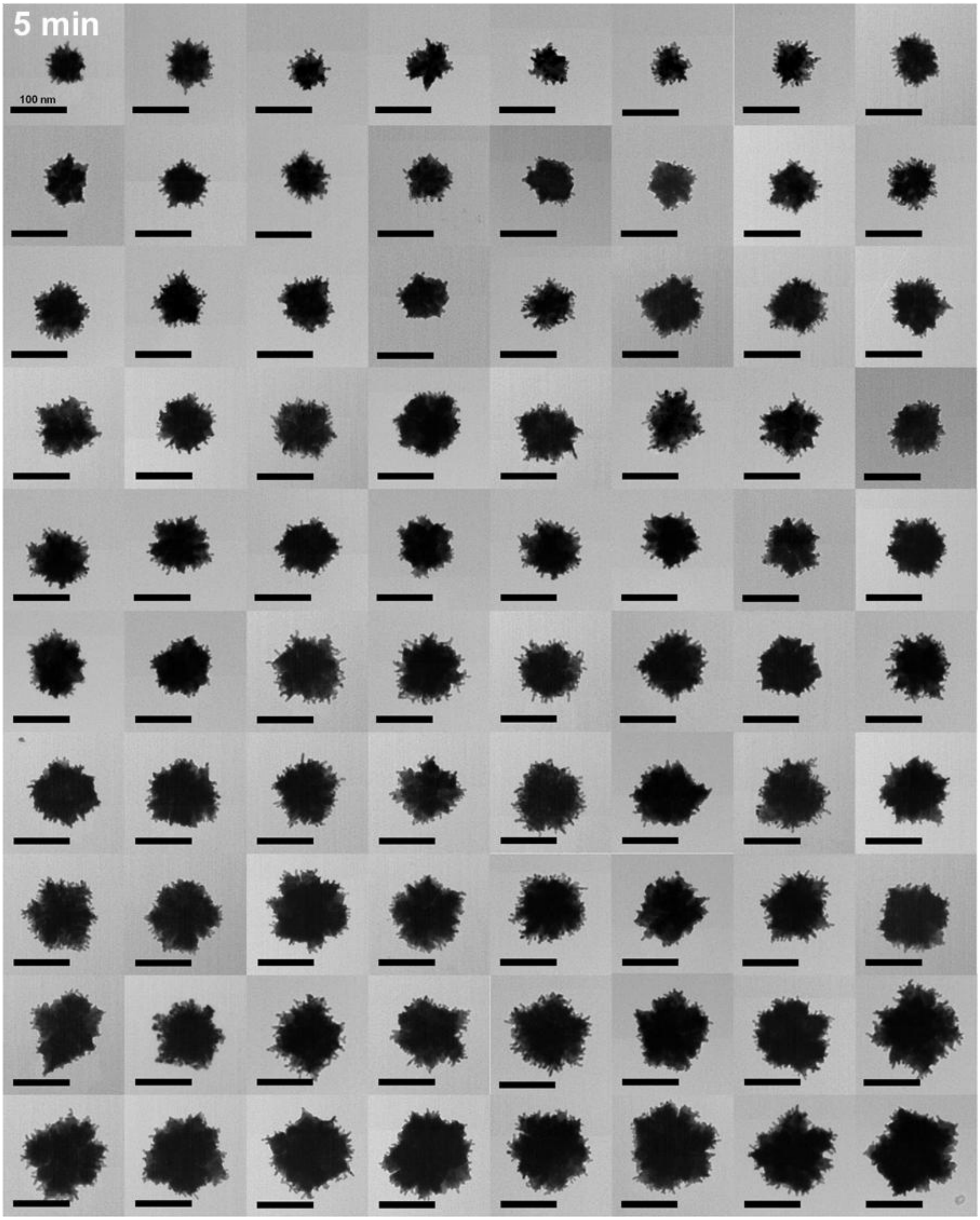
5 min
100 nm

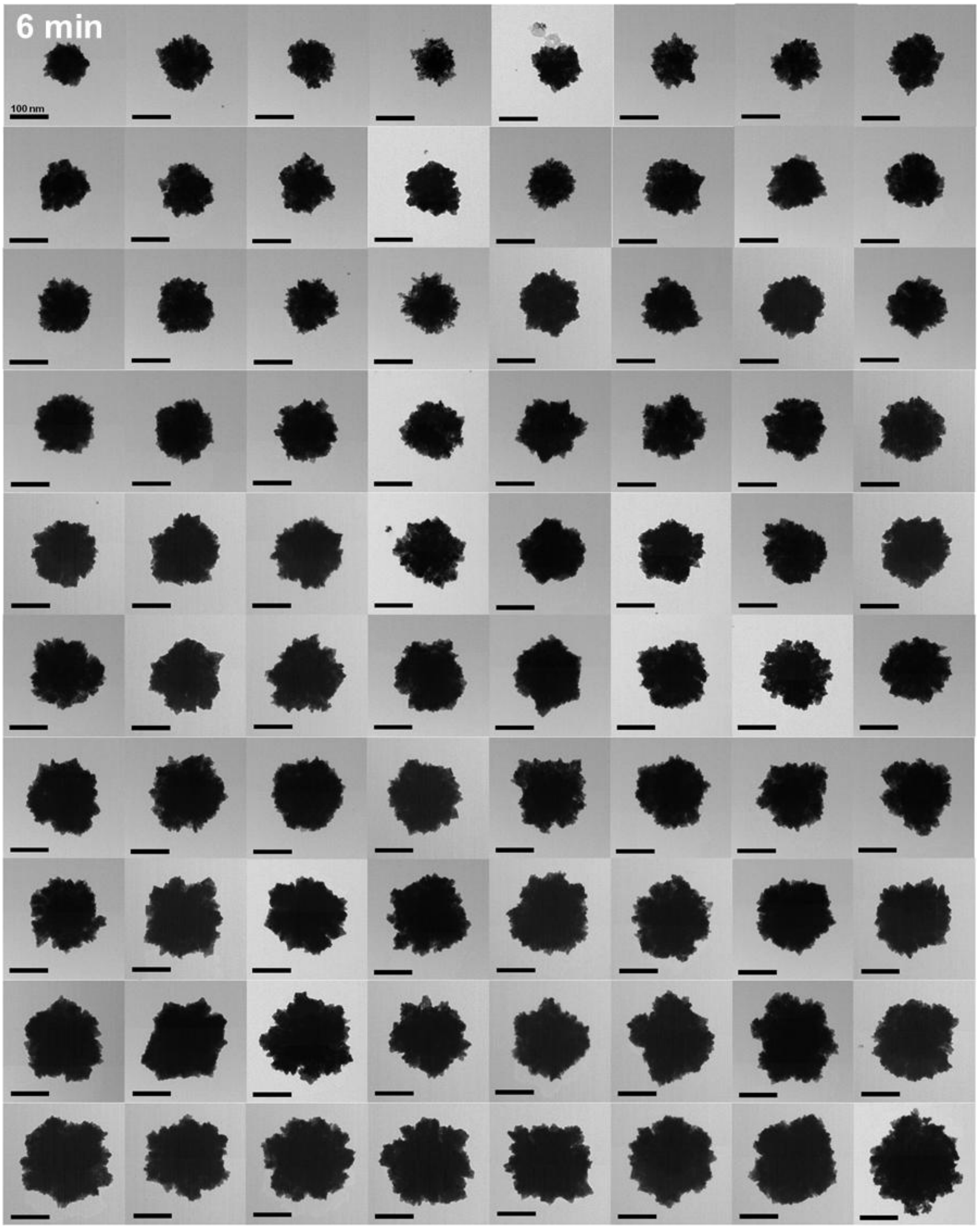
6 min
100 nm

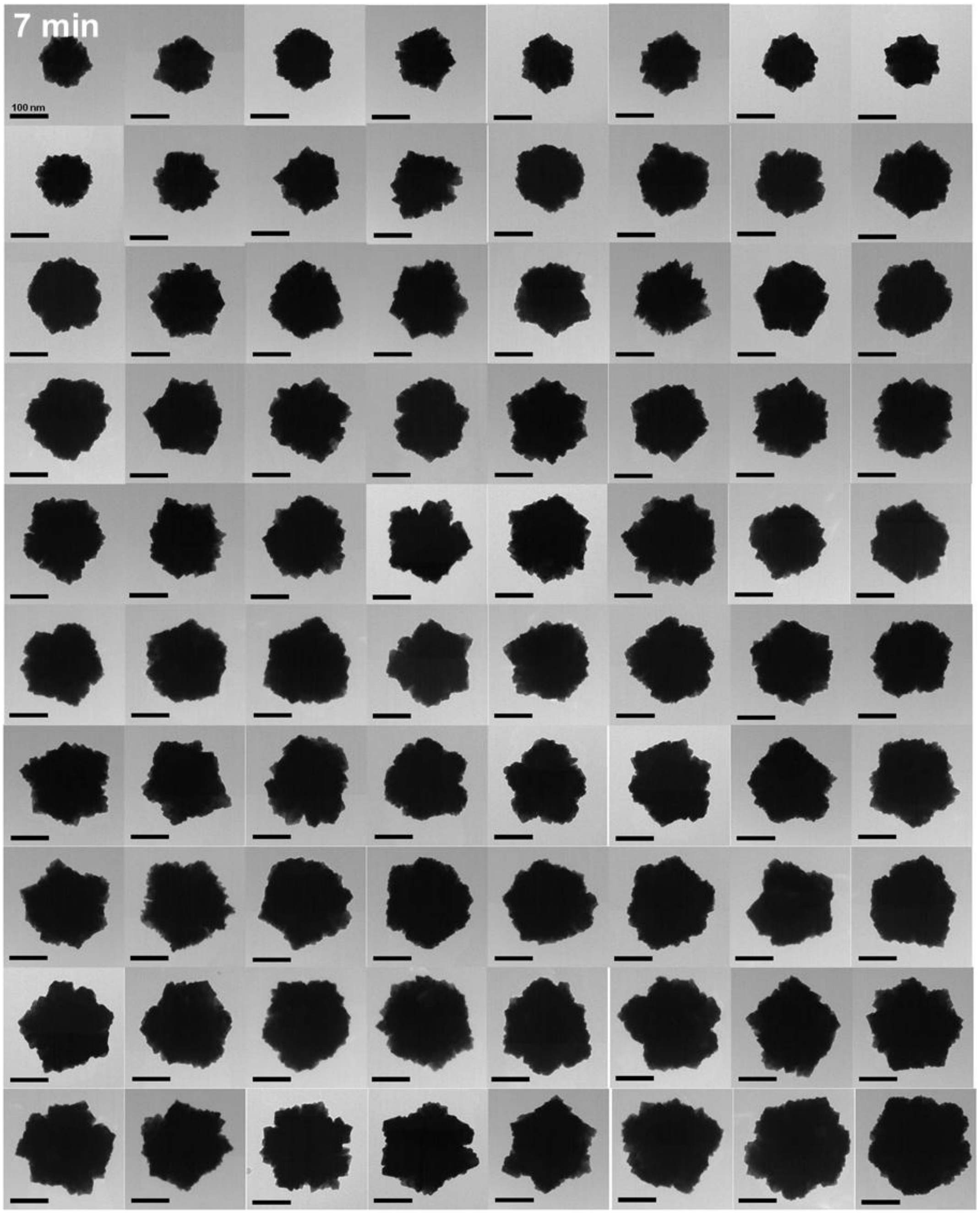
7 min
100 nm

b

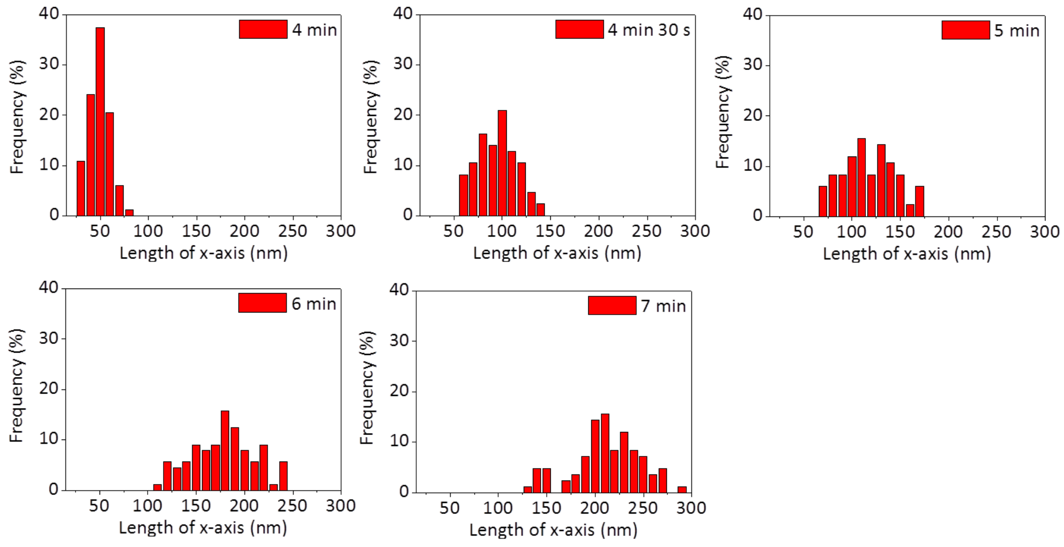
4 min
4 min 30 s
5 min
6 min
7 min
Frequency (%)
Length of x-axis (nm)
0
10
20
30
40
50
100
150
200
250
300

c

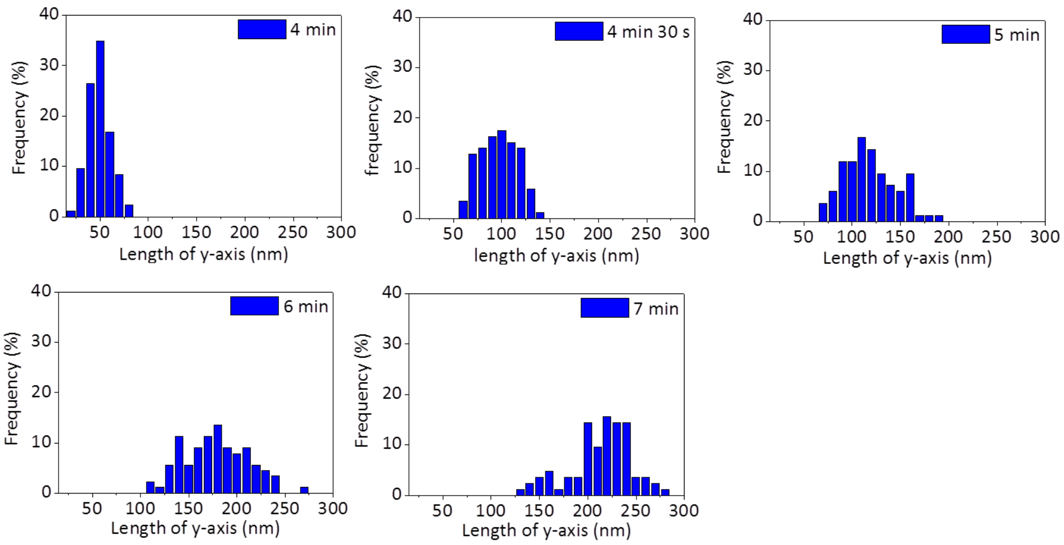
4 min
4 min 30 s
5 min
6 min
7 min
Frequency (%)
frequency (%)
Length of y-axis (nm)
length of y-axis (nm)
0
10
20
30
40
50
100
150
200
250
300

d

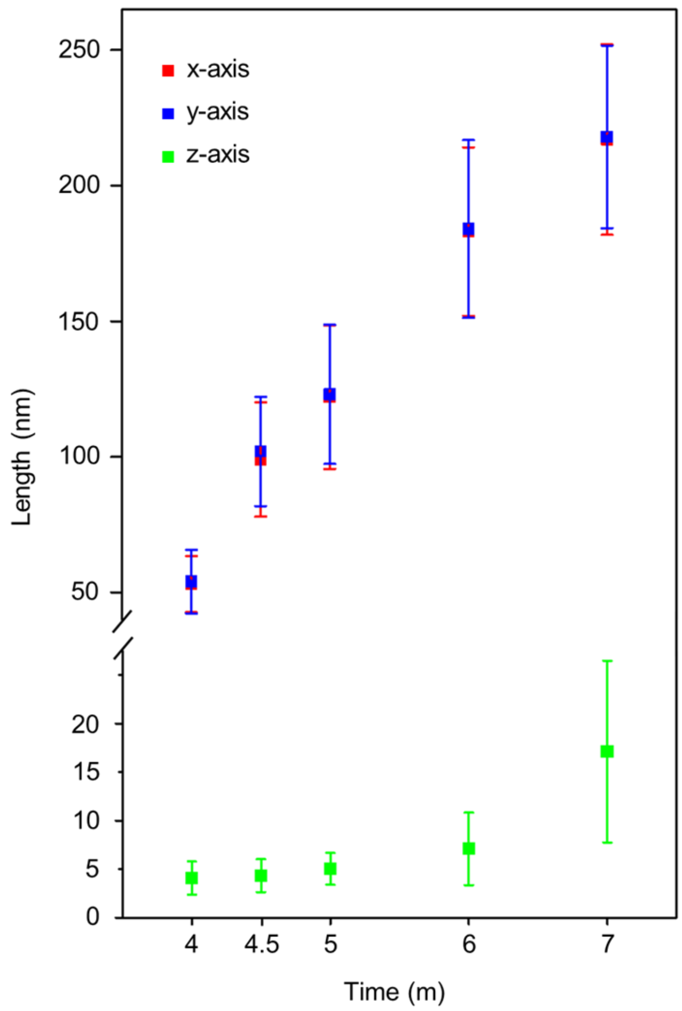

Supplementary Figure 1 | (a) Transmission electron microscopy (TEM) images of two-dimensional (2D) gold nanoparticles sampled at the oleic acid/water interface at 4 min, 4 min 30 s, 5 min, 6 min, and 7 min after addition of oleic acid on top of aqueous phase. (b) Lateral size distributions with respect to x-axis of the particles. (c) Lateral size distributions with respect to y-axis of the particles. For statistical analyses, 80 particles are randomly selected. (d) Average lengths with respect to x- (red), y- (blue) and z-axes (green) of the particles sampled at the interface with increasing reaction time.

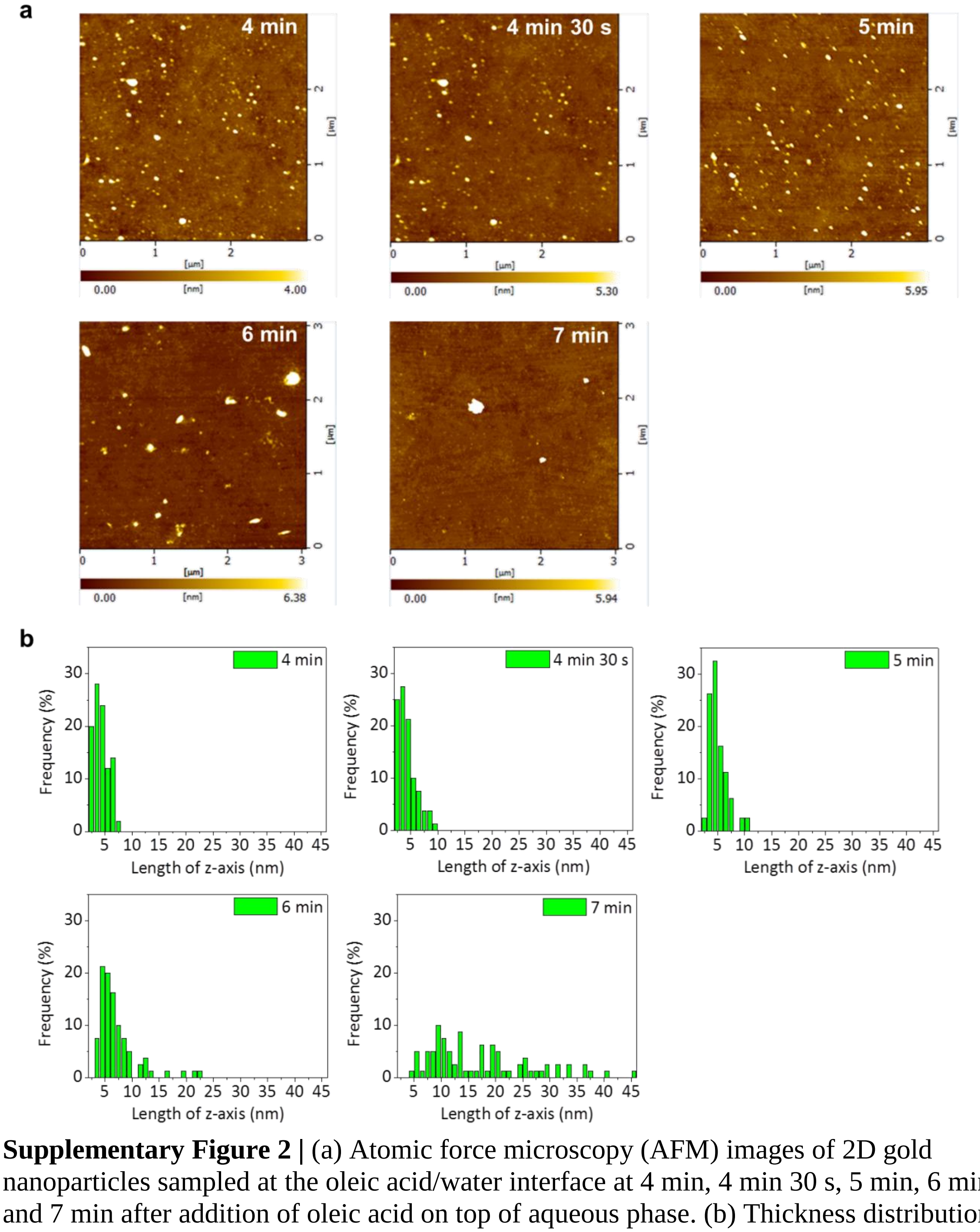

Supplementary Figure 2 | (a) Atomic force microscopy (AFM) images of 2D gold nanoparticles sampled at the oleic acid/water interface at 4 min, 4 min 30 s, 5 min, 6 min, and 7 min after addition of oleic acid on top of aqueous phase. (b) Thickness distributions with respect to z-axis of the particles.

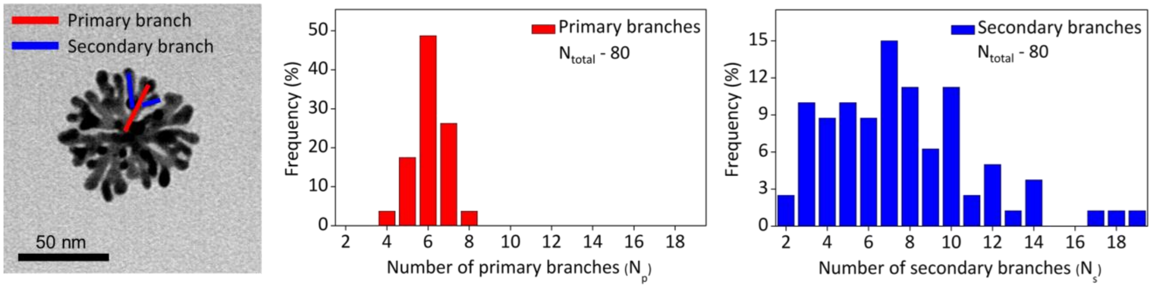

Supplementary Figure 3 | Statistical analyses for the number of primary and secondary branches of 2D gold nanodendrimers.

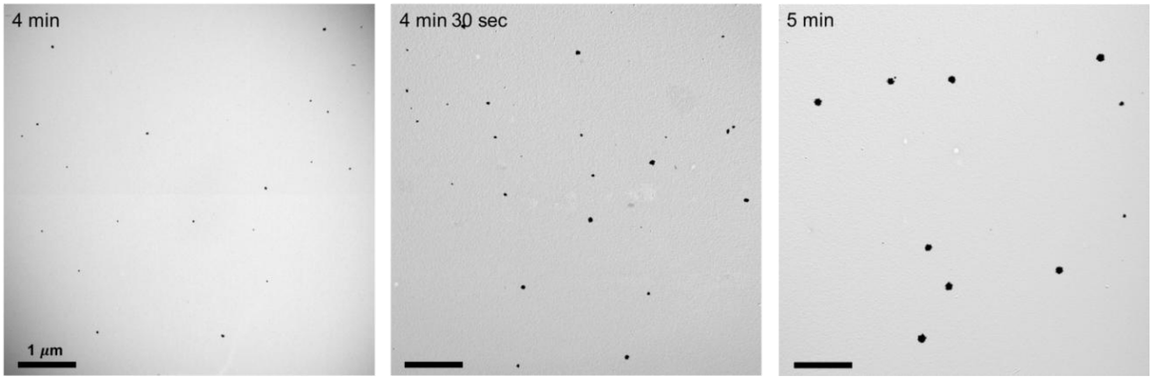

Supplementary Figure 4 | Transmission electron microscopy (TEM) images of 2D gold nanoparticle sampled at the oleic acid/water interface for 4 min, 4 min 30 s, and 5 min. Scale bars correspond to 1 μm.

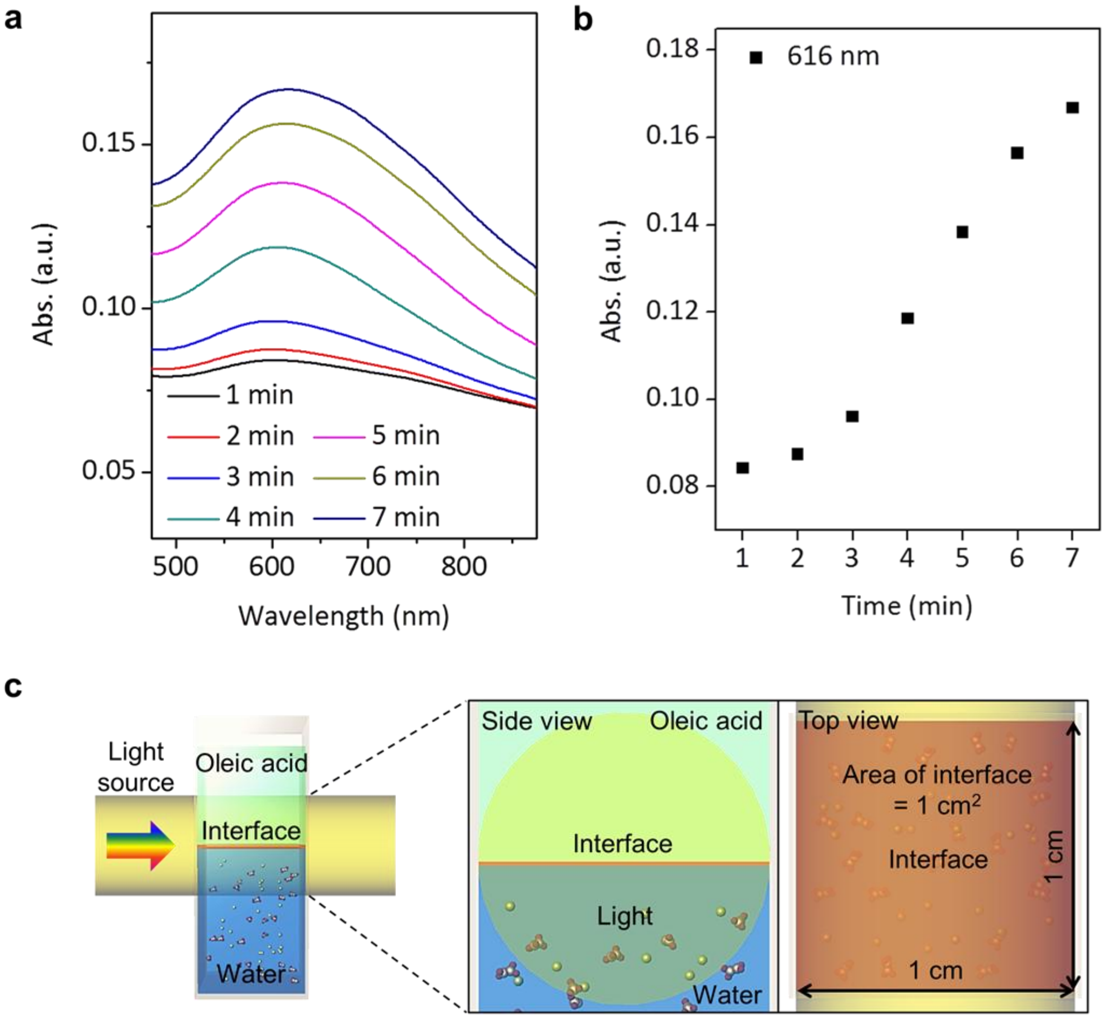

Supplementary Figure 5 | (a) Time-resolved UV-vis measurement at the oleic acid/water interface. The light source was selectively illuminated to the oleic acid/water interface. (b) Plot of absorbance at 616 nm as a function of time. (c) Cross-sectional area of the oleic acid/water interface illuminated by the light source. The yellow circle indicates the light source.

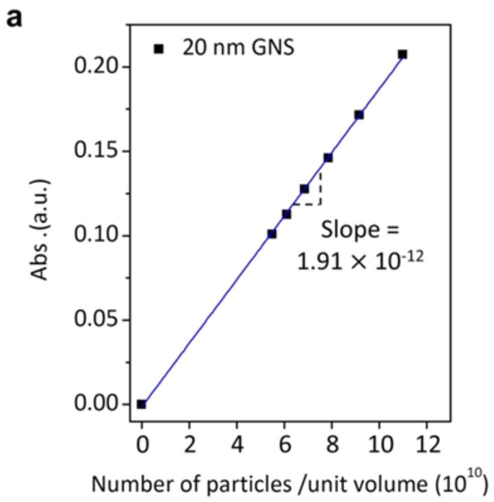

b

At oleic acid/water interface	Absorbance of a single particle (slope)	1.91×10^{-12}
	Δ Absorbance at peak position at 4 min (616 nm)	0.03545
	Number of particles per unit area (cm^2)	1.85×10^{10}

Supplementary Figure 6 | (a) Plot of the absorbance of 20 nm gold nanospheres (GNS) at different concentrations. (b) Estimation of the density of the particles distributed on the oleic acid/water interface.

	Acidic ← Less reactive						
Solution pH	3.05	3.26	3.47	3.51	4.02	4.72	6.28
Time of color change at interface	5 min	1 min 40 s	1 min	35 s	15 s	1 s	Color change both at interface and in aqueous phase
Optical image obtained at 60 sec	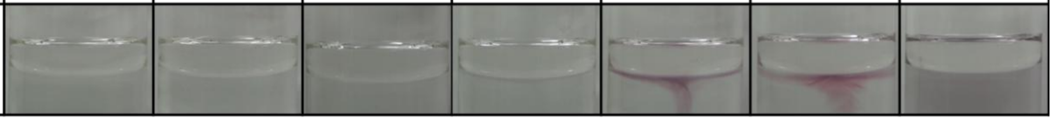						

Supplementary Figure 7 | Effect of solution pH on the formation of gold nanoaprticles at the oleic acid/water interface. Note that reducing agent is expected to exist mainly with deprotonated form (NH_2OH) at solution pH = 6.28.

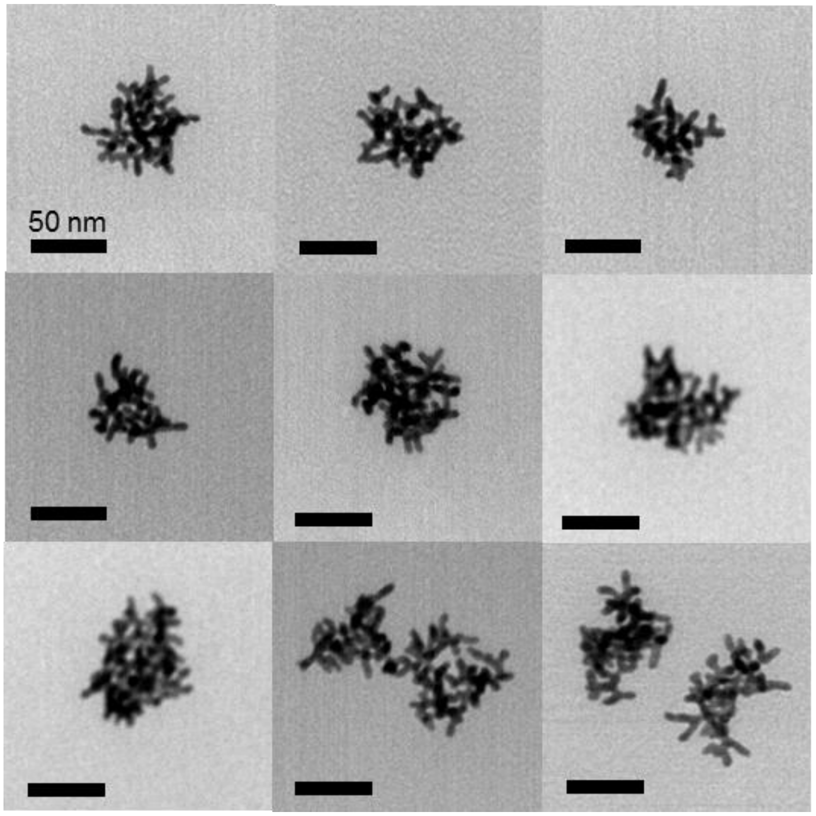

Supplementary Figure 8 | Representative TEM images of 2D gold nanoparticles synthesized in oleic acid-in-water emulsions.

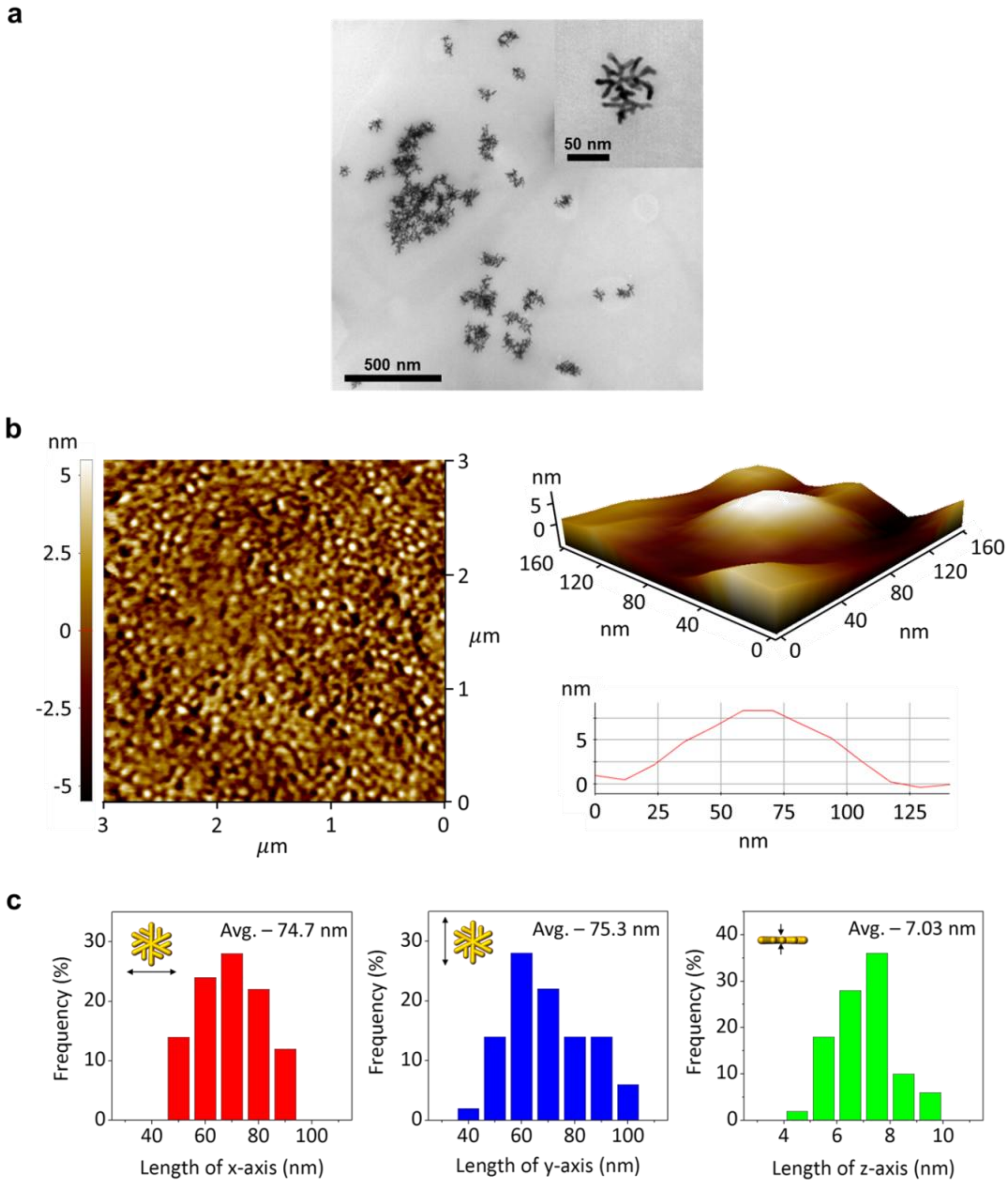

Supplementary Figure 9 | (a) TEM image of dendritic gold nanoparticles obtained from an emulsion. (b) Large-area atomic force microscopy (AFM) image and representative AFM image of the dendritic gold nanoparticles. (c) Size distributions and average lengths with respect to x- (red), y- (blue) and z-axis (green) of the particles. For statistical analyses, 50 particles are randomly selected.

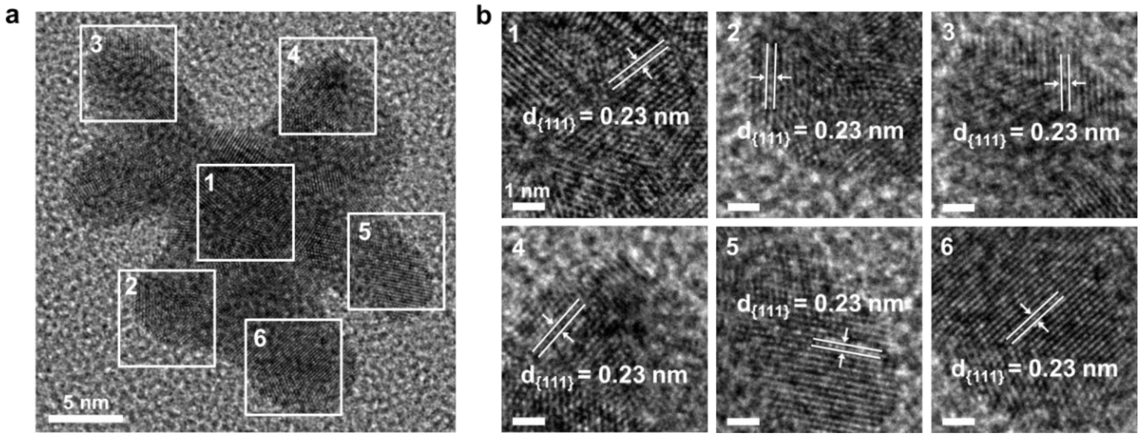

Supplementary Figure 10 | (a) High-resolution transmission electron microscopy (HRTEM) image of 2D dendritic gold nanoparticle obtained at the oleic acid/water interface. (b) Lattice-resolved images (numbered 1-6 boxes in (a)).

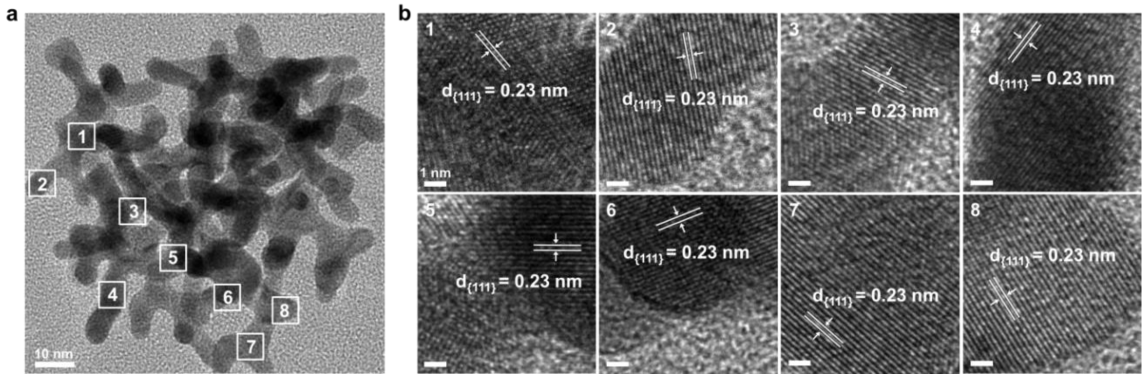

Supplementary Figure 11 | (a) HRTEM image of 2D dendritic gold nanoparticle obtained from the oleic acid-in-water emulsion. (b) Lattice-resolved images (numbered 1-8 boxes in (a)).

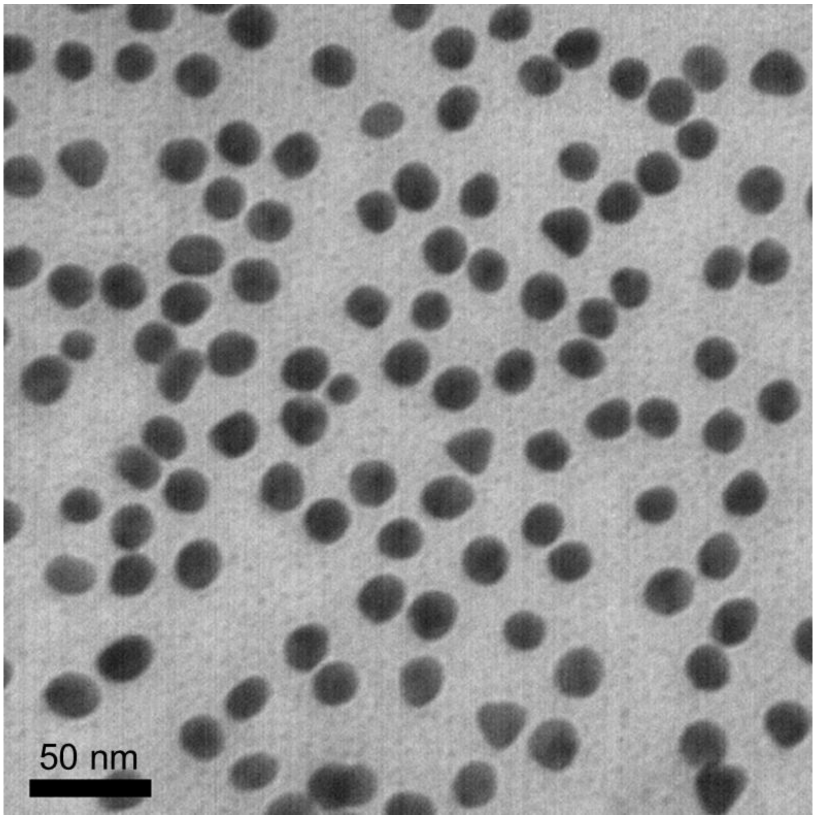

Supplementary Figure 12 | TEM image of gold nanospheres used for a surface-enhanced Raman spectroscopy (SERS) experiment.

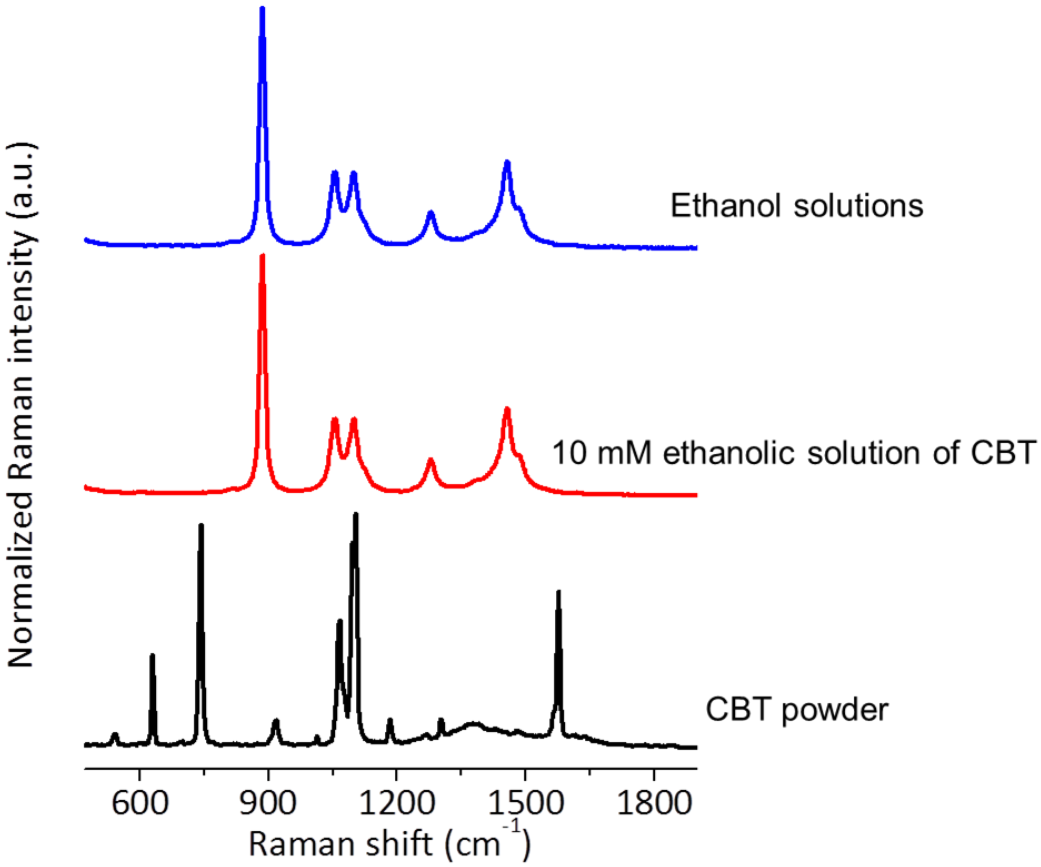

Supplementary Figure 13 | Intrinsic Raman spectra of 4-chlorobenzenethiol (CBT) powder, 10 mM ethanolic solution of CBT (Note that no apparent Raman transition of CBT is shown in the Raman spectrum) and ethanol solutions.

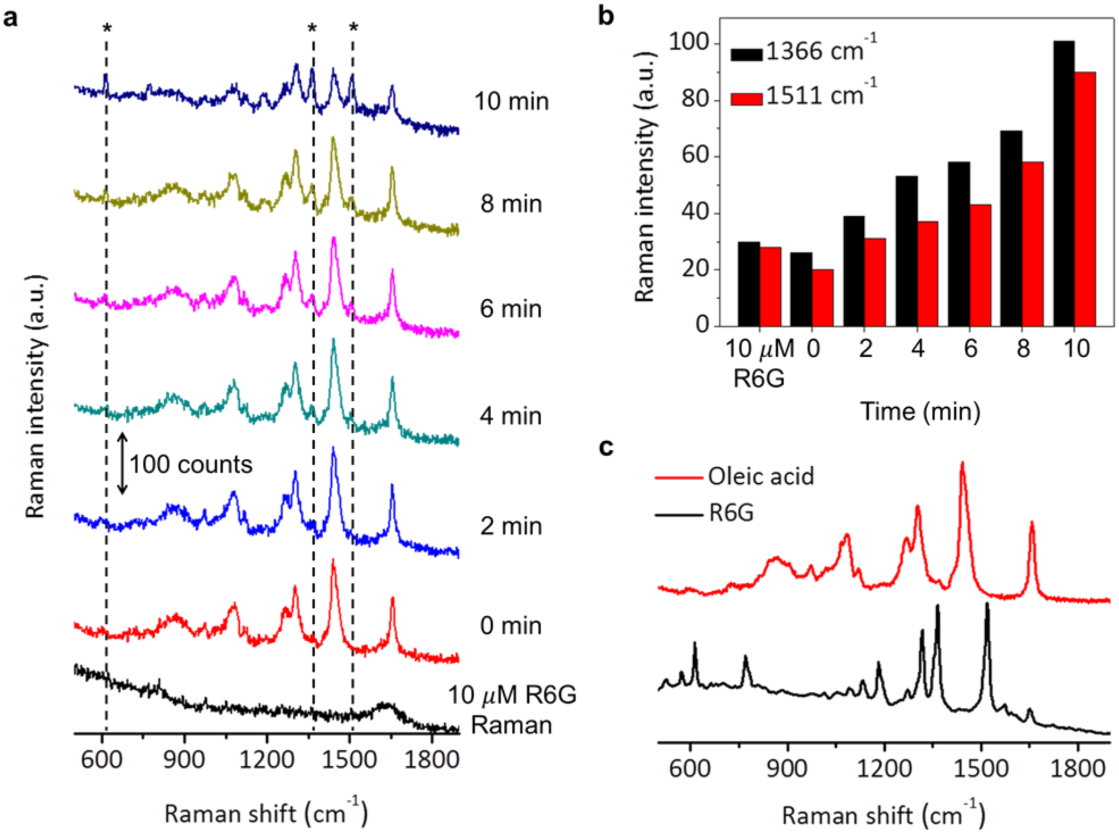

Supplementary Figure 14 | (a) Time-resolved SERS spectra measured from the oleic acid/water interface. The Raman transitions of R6G are marked with dash lines and asterisks (613, 1366, and 1511 cm^{-1}, respectively). (b) Comparison of the Raman intensity at the oleic acid/water interface as a function of time. (c) Intrinsic Raman spectra of oleic acid and R6G.

Particle	Sample concentration (ppm)	Volume per particle (nm^3)	Number of particles/50 μl
Gold nanodendrimer	11.98	9.82×10^3	3.16×10^{10}
20 nm gold nanosphere	4.49	4.19×10^3	2.78×10^{10}

Supplementary Table 1 | Concentrations of gold nanodendrimers and gold nanospheres measured from ICP-MS analysis.

	Standard solution	Gold nanodendrimer
Raman intensity at 1066cm^{-1} ($I_{solution}$ and I_{SERS})	5	429
Number of CBT ($N_{solution}$ and N_{SERS})	3.01×10^{18}	4.18×10^{15}
SERS enhancement factros	6.18×10^{3}	

Supplementary Table 2 | SERS enhancement factor for the experiment.

Supplementary Movie 1

Experimental demonstration for our oil/water interfacial synthesis of two-dimensional gold nanoparticles.

Supplementary Movie 2

Phase field crystal simulation showing the branching of the nucleus at the early stage of growth.
